# Functional Activities and Immunohistochemical Cellular Distribution of Glutathione S-Transferases in Normal, Dysplastic, and Squamous Cell Carcinoma Human Oral Tissues

**DOI:** 10.4137/cmo.s491

**Published:** 2008-03-28

**Authors:** Peter J. Giannini, Mark A. Morse, Christopher M. Weghorst, Ping Pei, Susan R. Mallery

**Affiliations:** 1 Department of Oral Biology, University of Nebraska Medical Center College of Dentistry, Lincoln, Nebraska; 2 Senior Toxicologist, Springborn Laboratories, Spencerville, Ohio; 3 Division of Environmental Health Sciences, School of Public Health, The Ohio State University, College of Medicine, Columbus, Ohio; 4 Department of Oral and Maxillofacial Surgery and Pathology, The Ohio State University, College of Dentistry, Columbus, Ohio; 5 The Ohio State University Comprehensive Cancer Center, Columbus, Ohio

**Keywords:** glutathione transferases, carcinogen metabolism, oral squamous cell carcinoma, dysplasia

## Abstract

Clinical data show a strong correlation between tobacco and alcohol use and the development of oral squamous cell carcinoma (SCC). While this association implies that the oral mucosa actively metabolizes carcinogens, there is little information which depicts the carcinogen metabolizing enzymes within the oral cavity. Glutathione S-transferases (GSTs) primary function is to detoxify carcinogens by increasing their water solubility, GSTs represent key carcinogen metabolizing enzymes. Notably, individuals with a null phenotype for certain GST isoforms are at an increased risk to develop cancer. This study investigated the function and distribution of GSTs in human oral tissues. Our results from this pilot study showed a trend towards higher GST activities in SCC tissues relative to normal mucosa. Also, relative to normal tissues, the SCC and epithelial dysplasia samples showed a more intense and uniform GST intracellular distribution. GST activities are increased in many high grade cancers. Similarly, our data suggest that GST upregulation occurs in at least a subset of precancerous and malignant oral lesions.

## Introduction

The development of oral SCC correlates strongly with the carcinogen exposure which occurs during tobacco and ethanol use (Neville et al. 2002; La Vecchia et al. 1997). Furthermore, development of oral SCC is thought to be a multistep process, with the initial phase of carcinogenesis entailing enzymatic bioactivation of the pro-carcinogen, followed by the covalent binding of the activated carcinogen to nuclear DNA (Harris, 1991). Although clinical evidence strongly supports the premise that the oral mucosa is actively engaged in carcinogen metabolism, little is known regarding the distribution and activities of carcinogen metabolizing i.e. Phase I and II enzymes, within the oral cavity.

Exposure to xenobiotics, such as tobacco associated carcinogens, induces expression and function of both Phase I (e.g. cytochrome P450s) and Phase II (e.g. GSTs) enzymes (Guengerich, 1991; Hayes et al. 1995). Although the microsomally located P450s are generally considered chemical detoxifiers, the P450s catalyze oxidation reactions that have the potential for carcinogen bioactivation. Glutathione S-transferases (GSTs) are a superfamily of Phase II enzymes which are present in either a soluble (Alpha, Mu, Pi, and Theta) or membrane-bound (microsomal), state (Hayes et al. 1995). Specifically, GSTs catalyze the conjugation of electrophilic, hydrophobic carcinogens with the reduced thiol of the glutathione (GSH) tripeptide; a detoxification reaction that increases the water solubility and facilitates excretion in the urine (Bongers et al. 1995). Furthermore, as GSTs also detoxify carcinogens which have been bioactivated by Phase I enzymes (Wilce et al. 1994), the relative intracellular distribution of P450’s and GSTs may be critical in determining the extent of DNA damage following carcinogen exposure.

Recently, studies have begun to characterize oral mucosal GST activities. Saroja et al. demonstrated that relative to histologically normal mucosa, tissue derived from Stage IV oral SCC tissues demonstrate significantly higher levels of total GST activities (Saroja et al. 1999). Similarly, Rawal et al. determined that relative to corresponding adjacent histologically normal tissues, oral SCC samples possess significantly higher GST levels (Rawal et al. 1999). Notably, neither of these previous reports determined GST expression in precancerous oral lesions. In addition, although these previous studies report higher GST functional activities in SCC relative to non neoplastic oral mucosal samples, these data do not refute the possibility of a biphasic role for GSTs in oral carcinogenesis. Low GST levels could enhance the mutation-associated initiation phase of carcinogenesis, while the acquisition of increased GST function by oral SCC lesions could facilitate tumor cell survival during cancer progression. This premise is supported by the fact that highly progressed tumors frequently overexpress cytoprotective enzymes, including GSTs, which partially account for some of their resistance to various chemotherapeutic agents (Saroja et al. 1999; Rawal et al. 1999).

Although characterizing the distribution of Phase I and II enzymes within the oral cavity is of scientific interest, these data could also directly impact translational applications regarding chemopreventive agent selection and use. The purpose of this study was to evaluate the cellular distribution of three GST isoforms (GST A1-1, GST M2-2, and GST P1-1) in normal, dysplastic, and oral SCC tissue. These specific isoforms were selected for evaluation due to the recognized association between polymorphisms in these isoforms and the development of human epithelial origin cancers (Their et al. 2003). GST A1-1 is the most abundant GST isoform (Ping et al. 2006). The GST Alpha isoform has been shown to confer protection against products of lipid peroxidation, such as α,β-unsaturated aldehydes that can be produced in response to various oxidative stimuli, for example, chronic alcohol exposure (Alin et al. 1985; Ingelman-Sundberg et al. 1984). In previous studies GST M2-2 has been shown to be effective in reducing benzo[a]pyrenedioleepoxide (BPDE) induced cellular DNA damage. BPDE represents a highly active metabolite of benzo[a]pyrene (B(a)P), a significant tobacco-associated carcinogen that can form DNA adducts leading to (B(a)p)-induced neoplasia (Shinozaki et al. 1998; Lee et al. 1998; Weng et al. 2005). GST-P1 is the isoform that is most associated with the detoxification of tobacco-associated carcinogens and it is also the most abundant GST enzyme in the head and neck area (Mulder et al. 1995). In addition, GST functional enzymatic analyses were conducted on both normal and SCC derived tissues. Our results suggest that at least a subset of premalignant and malignant oral lesions undergoes a biochemical adaptation resulting in increased GST levels.

## Materials and Methods

### Oral mucosal tissue samples

All tissues used for these studies were obtained in compliance with the stipulations of The Ohio State University’s Institutional Review Board for use of human tissues. Ten normal oral mucosal samples were obtained from patients undergoing elective oral surgical procedures at The Ohio State University College of Dentistry. At the surgical appointment, social and medical histories were obtained from the tissue donors, and clinical assessments (presence or absence of inflammation or surface ulceration), made of the tissue sites. All of the patients from which normal samples were obtained had noncontributory medical histories. One patient reported an allergy to penicillin. Subsequent to obtaining the necessary consent, normal samples were collected under local anesthesia at the time of the patient’s elective oral surgical procedure.

Fourteen samples of oral SCC were obtained from patients undergoing tumor resections at The Ohio State University James Cancer Hospital through The Ohio State University Tissue Procurement Network. Medical and social histories were provided by Tissue Procurement Network for the SCC tissue donors. Oral SCC samples were collected by tissue procurement services from patients undergoing surgical excision of their neoplasm. Other than their diagnosis of oral SCC, patients exhibited an otherwise unremarkable medical history with no previous history of radiation or chemotherapy treatments. Surgical specimens were received in Modified Eagle’s Medium transport media.

Normal and SCC samples were processed in the following manner: a representative sample was fixed for two hours in 10% neutral buffered formalin, followed by routine tissue processing and paraffin embedding. The remainder of the sample was snap frozen (−80 °C) until preparation of cytosolic extracts. Routine hematoxylin and eosin sections were prepared of all tissue samples to confirm the diagnosis, and to assess extent and character of any inflammatory cell infiltrate. Following microscopic confirmation of the clinical diagnosis, multiple 4 μm sections were prepared and placed on Superfrost/Plus slides (Fisher Scientific, Pittsburgh, PA) for immunohistochemical analyses.

In addition, 10 archival samples of formalin-fixed oral epithelial dysplasia (samples included mild, moderate, and severe dysplasia which had undergone a similar short-duration formalin fixation) were prepared for immunohistochemical analyses.

### Immunohistochemistry

Sections of normal, dysplastic, and SCC tissue were deparaffinized, and hydrated using standard techniques. Peroxidase activity was then blocked with 0.3% H_2_O_2_ in methanol for 30 minutes at room temperature, followed by a rinse with phosphate buffered saline (PBS). Following incubation with normal goat blocking serum for 20 minutes (Vector Laboratories, Burlingame, CA), and avidin and biotin blocking agents for 15 minutes each (Vector Laboratories, Burlingame, CA), sections were incubated with three GST antibodies (Vector Laboratories, Burlingame, CA.) GST A1-1 (1:100), GST M2-2 (1:200), and GST P1-1 (1:100) overnight at 4 °C in a humidified chamber. Sections were then incubated with biotinylated secondary antibody for 30 minutes (Vector Laboratories, Burlingame, CA), rinsed with PBS, and then incubated with the VECTASTAIN ABC Elite kit for 30 minutes (Vector Laboratories, Burlingame, CA). Next, the slides were rinsed with PBS, incubated with DAB (3,3′-diaminobenzidine) substrate (Vector Laboratories, Burlingame, CA), rinsed in water, counterstained with hematoxylin, dehydrated, and mounted with Permount (Fisher Scientific, Pittsburgh, PA.). The isotypic negative controls received identical handling, with the exception that pre-immune blocking serum (Vector Laboratories, Burlingame, CA) was used instead of primary antibody.

### Preparation of cytosols for functional GST analyses

Tissue fragments (10–100 mg) were thawed and homogenized on ice in 5 volumes of homogenization buffer (0.05 M Tris-HCl, 1.15% KCl, and 0.1 mM phenylmethylsulfonyl fluoride (PMSF), pH 7.4 using a Polytron homogenizer (Kinnematica, Switzerland). The homogenates were centrifuged at 150,000 × g at 4 °C for 1 hour. The volume of the cytosolic extract was recorded, and a representative aliquot taken for protein determination, and the supernates stored at −80 °C until analysis of GST functional activity was performed.

### Determination of total GST functional activity

The method of (Habig et al. 1974) was used to determine tissue total GST functional activity. GST activity was spectrophotometrically determined (Beckman DU 7400 equipped with a Peltier temperature regulator) by measuring the formation of the conjugation product of chloro-dinitrobenzene (CDNB) and reduced GSH (Sigma Biochemical, St. Louis, MO) under the following assay conditions: 340 nm, pH 6.5, 1 mM reduced glutathione (GSH), 25 °C, 1.0 mM CDNB. With every assay, a concurrent five point standard curve (0.1, 0.5, 1.0, 5.0, 10.0 units/ml) was conducted, and specimen GST activities remained within the linear range of the standard curve. GST activity is expressed as units/mg protein, with 1 unit of GST defined as the enzymatic concentration that will conjugate 1.0 μmole of CDNB with reduced GSH per minute at pH 6.5 at 25 °C. CDNB is a universal substrate for Alpha, Mu and Pi GST isoforms. Results are expressed as units of activity per mg of sample protein.

### Protein determination

Sample protein levels were determined by using the BCA (bicinchoninic acid) Protein Assay Kit using bovine serum albumin as the standard protein (Pierce, Rockford, IL).

### Western blotting to confirm specificity of GST antibodies

Western blot analysis, using standard techniques, was performed to determine if cross-reactivity existed among the GST Alpha, Mu, and Pi antibodies. GST Alpha, Mu, and Pi proteins (Sigma Chemical Company, St. Louis, MO) were applied to sodium dodecyl sulfate polyacrylamide gels (SDS-PAGE), 30 μg of protein per well, which was run at 120 V for 1–2 hours. Following electrophoresis, the proteins were transferred to a Hybond-P PVDF membrane by placing the SDS-PAGE in direct contact with the PVDF membrane, which was then run at 100 V for 1 hour in a transfer buffer. Nonspecific binding of antibody was blocked with 5% nonfat milk in PBS + 0.5% Tween-20 (PBS-T) for 1 hour. Gels were then incubated with each of the three primary antibodies (GST α, μ, and π) in 5% nonfat milk in PBS-T overnight at 4 ^o^C. This was followed by incubation for 1 hour with a goat anti-rabbit secondary antibody conjugated to horseradish peroxidase in 5% nonfat milk in PBS-T at a dilution of 1:15,000. The gels were then incubated for 1 minute in detection solution at room temperature. Excess detection solution was drained from the blots. They were subsequently exposed to Kodak film in a darkroom, which was later developed to allow for visualization of the bands.

### Statistical analysis

A Mann-Whitney rank sum test was utilized to assess any significance in the observed GST functional activities between normal and oral SCC mucosa.

## Results

### Patient data and social history

Of the ten patients from whom samples of normal oral mucosa were obtained, the average age was 41 yrs. with a standard deviation of 20.3 yrs., and a 3:2 male to female ratio. None of the patients reported a personal or family history of cancer. In terms of race, 9 of 10 normal subjects were Caucasian and one was African-American. Four of the subjects had a significant smoking history, while none had a positive history of alcohol use. Dysplastic tissue samples were obtained from a variety of sites including vestibule, lip, tongue, palate, floor of mouth, and buccal mucosa. Most patients did not report a personal or family history of cancer. The average age was 53 yrs. with a standard deviation of 14.4 yrs., and a 7:3 male to female ratio. Similar to the patient group from whom normal oral mucosa was obtained, 9 of 10 patients in the dysplasia group were Caucasian and one was African-American. Most (70%) did have a history of smoking however, a significant alcohol history was not observed. Oral SCC tissue was harvested from a variety of sites including tongue, palate, tonsil, pharynx, buccal mucosa, and neck. The average age of oral SCC patients was 55 yrs. with a standard deviation of 13.5 yrs., and a 3:2 male to female ratio. All of the oral SCC patients were Caucasian. Most patients reported no prior personal or family history of cancer however, 67% had reported a significant smoking history. Likewise, 50% of the patients had a significant alcohol consumption history.

### Immunohistochemical analysis of GST Alpha, Mu, and Pi

**Figure 1:**
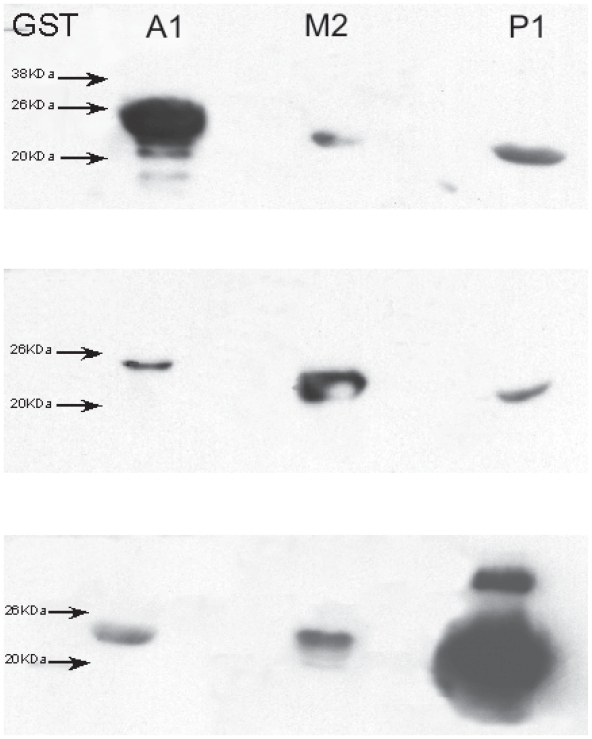
Western blot analysis for GST A1-1, M2-2, and P1-1 demonstrating GST antibody specificity.

In order to verify that there was no cross-reactivity between the GST antibodies, a Western Blot was performed (Fig. 1). The immunoblot results demonstrate a lack of cross-reactivity and show that the GST antibodies (A1-1), (M2-2), and (P1-1) were specific for their respective antigen.

**Figure 2:**
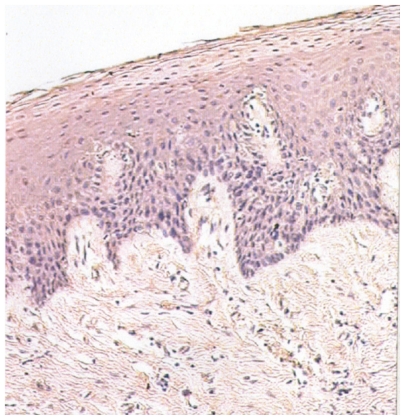
GST A1-1 expression in normal oral mucosa (100X). Mild GST expression is primarily localized to the cytoplasm of cells within the basal layer.

**Figure 3:**
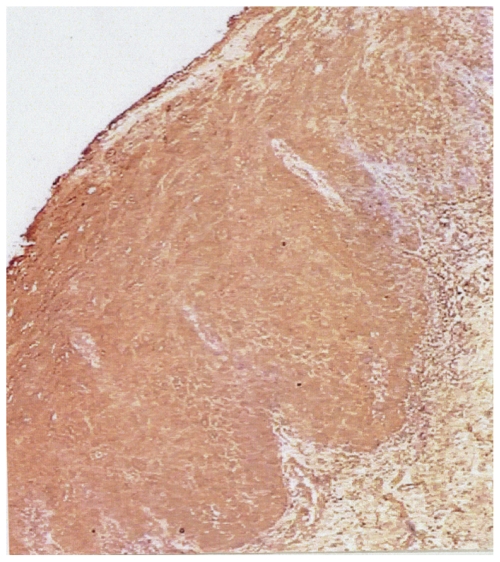
GST P1-1 expression in dysplastic oral mucosa (100X). Moderate GST expression is demonstrated throughout the entire epithelium.

**Figure 4:**
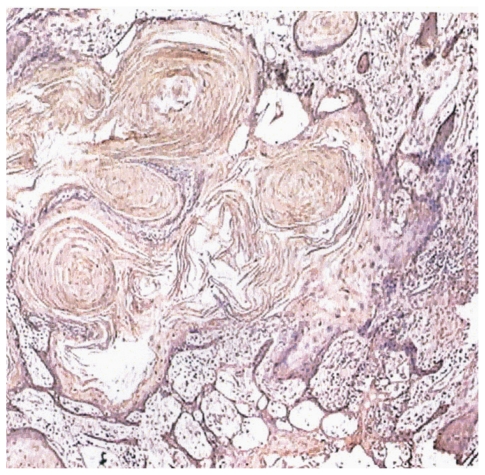
GST P1-1 expression in well-differentiated oral SCC (40X). Moderate GST expression is observed to be more intense within the basal cells of the invading epithelial islands.

Immunoreactivity of parraffin-embedded oral tissue using GST (A1-1), (M2-2), and (P1-1) polyclonal antibodies showed differential expression of these proteins in normal, dysplastic, and oral SCC tissues. Representative oral tissue immunohistochemical sections are shown in (Figs. 2–4). A common finding among all tissues analyzed was greater staining, corresponding to increased GST expression in the surface epithelial cells relative to connective tissues. The surface epithelium of normal tissues demonstrated a light to moderate GST staining for the Alpha, Mu, and Pi isoforms; findings that are indicative of a low level of expression of each of the three GST proteins that were investigated. In normal epithelium, GST proteins appear to be localized primarily within the cytoplasm of cells in the basal layer, with decreased expression apparent in the more differentiated epithelial cells. In contrast, dysplastic oral tissues (regardless of histologic grade) did not manifest the differentiation-associated GST expression. Dysplastic samples showed moderate expression of GST proteins throughout the epithelium. Intense expression of GST Alpha, Mu, and Pi was observed in all oral SCC tissues. Well-differentiated tumors demonstrated moderate expression of all three GST isoforms, with greater expression in the basal cells of the invading epithelial islands, similar to that observed in normal mucosa. Greatest GST expression, however, was detected in the highest grade tumors, corresponding to 5/14 or 36% of oral SCC samples. Also unique to the SCC samples was preferential expression of the GST π isoform.

### GST enzyme activity

Cytosolic preparations from both normal and oral SCC samples were prepared for the determination of the units of GST enzymatic activity per milligram of protein (GST U/mg) (Table 1). We did not discern any correlations between GST functional activities and the presence or absence of inflammation, or use of either alcohol and/or tobacco.

Comparison of the GST enzymatic activities of normal and SCC oral mucosa revealed a trend toward higher GST enzyme activity for the SCC samples (Table 1). In addition, consistent with the recognized extensive heterogeneity in human Phase II enzyme activities, a wide range of GST functional activities was detected in our specimens. While the histologically normal tissues demonstrated a 93 fold difference in GST activities, an even greater range (107 fold difference in GST activities) was detected among the SCC samples. In comparing the mean GST activities of the normal and oral SCC samples, a 2.5 fold increase in mean GST activity was noted in the SCC specimens. Results from a Mann-Whitney rank sum test for the comparison of GST enzymatic activities of normal and SCC oral mucosa did not reveal a statistically significant difference in these values at the p = 0.05 level of significance.

## Discussion

The GSTs are part of a complex supergene family of detoxification proteins which are involved in conjugation reactions which increase the water solubility of electrophilic compounds, thus facilitating their elimination (Hayes et al. 1995). GSTs exist in five main classes: Alpha, Mu, Pi, Theta, and microsomal or membrane-bound. The GSTs’ cytoprotective effects extend beyond primary carcinogen metabolism. In the event that a procarcinogen is bioactivated to a carcinogen by phase I enzymes, such as the cytochrome P450s, GSTs can subsequently detoxify the agent prior to cell damage.

It is well known that GST polymorphisms can modify the gene expression of GST proteins. Previous studies have been conducted to investigate the role of GST null genotypes, specifically the GST M1-1 null genotype, and its relationship to the risk for such cancers as lung, bladder, and oral SCC (Rebbick, 1997). The prevalence of the GST M1-1 null genotype averages 50% in various populations including persons of European, Hispanic, and Asian descent. In persons of African descent the prevalence of the GST M1-1 null genotype is about 40%. Previous studies have demonstrated that the GST M1-1 null genotype does not impart a significantly increased risk for the development of oral SCC (Park et al. 1997; Hung et al. 1997; Jourenkova-Mironova et al. 1999; Gronau et al. 2003). Another study investigated the role of GST M1-1 polymorphisms and risk for oral SCC in African-Americans and Caucasians. In Caucasians the GST M1-1 null genotype revealed similar results imparting no increased risk for oral SCC. Conversely, in African-Americans, the GST M1-1 null genotype has been shown to significantly increase the risk for oral cancer (Park et al. 2000).

Our results from this pilot study showed minimal expression of all three GST isoforms, GST (A1-1), (M2-2), and (P1-1) in normal mucosa, with staining largely restricted to the cytoplasm and perinuclear regions of the basal cells. Most of the normal mucosal samples for this study were gingival (8/10) while the remaining samples were obtained from alveolar mucosa. The normal mucosal samples were harvested from patients undergoing elective oral surgical procedures. Some patients were undergoing surgery for third molar extractions while others were presenting for biopsies of lesions involving the gingiva or alveolar mucosa.

In contrast, there was markedly greater staining intensity observed in both dysplastic and SCC tissues. Six oral SCC samples were harvested from the tongue, two each were from the pharynx and cervical lymph nodes, with one each being harvested from the mandibular alveolar mucosa, buccal mucosa, soft palate, and tonsil.

Unlike the normal tissues, GST positive staining was observed throughout the epithelium in the dysplastic and SCC samples. This observation was most apparent with regard to the GST Pi isoform. Notably, GST Pi is the predominant isoform present in tissues of the gastrointestinal tract, lung, and erythrocytes (Tsuchida et al. 1989; Moscow et al. 1989). Relevant to our results, overexpression of the GST Pi isoform has been found in a variety of premalignant and malignant epithelial lesions. Previous studies have noted an increased expression of GST Pi in human premalignant and malignant tissues of the colon, kidney, and lung when compared to their surrounding normal counterparts (Chasseaud, 1979; Kodate et al. 1986; Di Ilio et al. 1987; Di Ilio et al. 1988). Our results also show greater staining intensity of GST Alpha, Mu, and Pi in more poorly differentiated relative to well-differentiated SCC tumors. Our pilot study data compare favorably to findings reported by Rawal et al. who observed that the more poorly differentiated SCC tumors possessed higher GST activities (Rawal et al. 1999). Several studies have provided mechanistic insights into how increased GST activities could provide a survival advantage to human cancers (Paumi et al. 2001; Goto et al. 2001). These selective survival advantages included rapid detoxification and transport of chemotherapeutic agents, as well as prevention of chemotherapeutic agent mediated DNA mutations (Harbottle et al. 2001; Sato et al. 1999).

Our data, which demonstrate large ranges in GST activities among the human donors, are consistent with the recognized heterogeneity in Phase I and II enzyme function in humans (Nebert et al. 1996). While our functional assays only showed a tendency towards higher GST function in SCC tumors, our immunohistochemistry results confirmed higher GST expression of all three isoforms evaluated in both the epithelial dysplasia and SCC lesions. Our results also suggest that GST expression manifests both a cell type and a growth state specificity, with highest levels of expression detected in proliferating epithelial cells. Because DNA synthesis is one of the most energetically costly cell processes, there is a concurrent increase in cellular oxidative metabolism and subsequent generation of reactive oxygen species and electrophilic molecules. Therefore, the observed increased GST expression in cells and tissues with high proliferation indices likely reflects a cytoprotective, reactive species and electrophile scavenging, cellular response.

The role of Phase I and II enzymes in either the induction or prevention of oral cancer is complex and entails interactions that include extent and type of carcinogen exposure, relative distribution and function of Phase I and II enzymes, and individual capacity to upregulate detoxification pathways. As GSTs serve a cytoprotective function, one could speculate that highest levels would be present in healthy, normal tissues. Therefore, detection of increased GST expression in premalignant and malignant lesions as noted in our and others’ studies may initially appear counter-intuitive. These collective data, however, imply that GST overexpression may facilitate cellular transformation by providing survival advantages in at least a subset of premalignant and malignant oral lesions. Recognition of unique biochemical features, such as GST overexpression, could facilitate subsequent development and selective targeting of chemopreventive and chemotherapeutic agents for premalignant and malignant oral lesions.

## Figures and Tables

**Table 1:** GST functional activities in normal and SCC tissues.

Normal mucosa + site	GST activity (U/mg)	Oral SCC + site	Histologic grade	GST activity (U/mg)
1 Gingiva	0.55	1 Tongue	Well differentiated	0.24
2 Gingiva	3.88	2A Tongue	Well differentiated	0.70
3 Gingiva	1.44	2B Lymph node with tumor	Well differentiated	0.77
4 Gingiva	2.50	2C Tongue	Well differentiated	1.25
5 Alveolar mucosa	4.67	3 Tonsil	Well differentiated	0.61
6 Gingiva	0.18	4 Tongue	Moderately differentiated	0.70
7 Gingiva	0.31	5 Cervical lymph node with tumor	Moderately differentiated	4.71
8 Alveolar mucosa	0.05	6 Mandibular mucosa	Well differentiated	0.83
9 Gingiva	0.23	7 Soft palate	Well differentiated	25.68
10 Gingiva	0.09	8 Tongue	Moderately differentiated	7.27
		9 Pharynx	Moderately differentiated	0.72
		10 Pharynx	Well differentiated	1.49
		11 Buccal mucosa	Well differentiated	1.76
		12 Tongue	Moderately differentiated	0.77
**Normal tissue mean ± s.e.m.** **1.39 ± 0.54**		**Oral SCC tissue mean ± s.e.m.** **3.43 ± 1.93**		

**Table 2:** GST functional activities, age, sex, smoking, and significant alcohol history in normal and SCC oral tissues.

Normal mucosa			
Age/Sex	GST activity (U/mg)	Smoking history	Alcohol history
46/M	0.55	Yes (1ppd/15yrs.)	None
23/F	3.88	None	None
50/M	1.44	None	None
22/M	2.50	Yes	None
78/F	4.67	None	None
69/M	0.18	None	None
22/M	0.31	None	None
51/F	0.05	Yes (1ppd/10yrs.)	None
22/M	0.23	None	None
22/F	0.09	Yes (1pack/week)	None

Oral SCC			
Age/Sex	GST activity (U/mg)	Smoking history	Alcohol history
54/F	0.24	None	None
50/M	0.70	Yes (60 pack yr.)	Heavy use
50/M	0.77	Yes (60 pack yr.)	Heavy use
50/M	1.25	Yes (60 pack yr.)	Heavy use
40/F	0.61	Yes (24 pack yr.)	Weekends only
54/F	0.70	None	None
30/M	4.71	Yes	3 drinks/day
42/F	0.83	None	None
66/M	25.68	Yes (42 pack yr.)	Previous alcohol abuse
65/M	7.27	None	None
56/M	0.72	Yes (80 pack yr.)	Quit 2 years prior
50/M	1.49	Yes	None
78/M	1.76	Yes	None
74/M	0.77	Yes	None
